# Strain-specific interspecies interactions between co-isolated pairs of *Staphylococcus aureus* and *Pseudomonas aeruginosa* from patients with tracheobronchitis or bronchial colonization

**DOI:** 10.1038/s41598-022-07018-5

**Published:** 2022-03-01

**Authors:** Meissiner Gomes-Fernandes, Andromeda-Celeste Gomez, Marc Bravo, Pol Huedo, Xavier Coves, Cristina Prat-Aymerich, Isidre Gibert, Alicia Lacoma, Daniel Yero

**Affiliations:** 1 Microbiology Department, Hospital Universitari Germans Trias i Pujol, Institut d’Investigació en Ciències de la Salut Germans Trias i Pujol, Universitat Autònoma de Barcelona (UAB), Badalona, Spain; 2 Institut de Biotecnologia i de Biomedicina (IBB), UAB, Barcelona, Spain; 3 CAPES Foundation, Ministry of Education of Brazil, Brasília, Brazil; 4 Departament de Genètica i de Microbiologia, UAB, Barcelona, Spain; 5 CIBER Enfermedades Respiratorias, CIBER, Instituto de Salud Carlos III, Badalona, Spain; 6 Julius Center for Health Sciences and Primary Care, University Medical Center Utrecht, Utrecht University, Utrecht, The Netherlands

**Keywords:** Bacterial pathogenesis, Biofilms, Pathogens, Clinical microbiology

## Abstract

Dual species interactions in co-isolated pairs of *Staphylococcus aureus* and *Pseudomonas aeruginosa* from patients with tracheobronchitis or bronchial colonization were examined. The genetic and phenotypic diversity between the isolates was high making the interactions detected strain-specific. Despite this, and the clinical origin of the strains, some interactions were common between some co-isolated pairs. For most pairs, *P. aeruginosa* exoproducts affected biofilm formation and reduced growth in vitro in its *S. aureus* counterpart. Conversely, *S. aureus* did not impair biofilm formation and stimulated swarming motility in *P. aeruginosa*. Co-culture in a medium that mimics respiratory mucus promoted coexistence and favored mixed microcolony formation within biofilms. Under these conditions, key genes controlled by quorum sensing were differentially regulated in both species in an isolate-dependent manner. Finally, co-infection in the acute infection model in *Galleria mellonella* larvae showed an additive effect only in the co-isolated pair in which *P. aeruginosa* affected less *S. aureus* growth. This work contributes to understanding the complex interspecies interactions between *P. aeruginosa* and *S. aureus* by studying strains isolated during acute infection.

## Introduction

The concern about polymicrobial infections has been increasing, especially regarding the biofilm environment, where *Staphylococcus aureus* and *Pseudomonas* aeruginosa, members of the microbiota and potential pathogens, interact with each other^1^. Further, an important number of human infections are associated with biofilms development, especially those associated with indwelling medical devices such as orotracheal tubes and urinary catheters^1,2^. It has been postulated that the most common lifestyle adopted by bacteria in nature is to grow in biofilm mode where the microbial community protects itself from antimicrobial agents, including both drugs and host defenses. Indeed the biofilm environment favors the development of antimicrobial resistance through a variety of mechanisms^3^ and thus facilitates the emergence of multidrug resistant (MDR) microorganisms.

*Staphylococcus aureus* and *P. aeruginosa* are two of the major MDR nosocomial pathogens^4^ commonly associated with lower respiratory tract infections (LRTI) in diverse clinical settings^5–7^. Both microorganisms are among the most common in the pulmonary microbiota displaying a range of cooperative and competitive interactions, and their co-isolation has been observed in a wide range of airway diseases^8^. In biofilm-related infections, such as the ones occurring in cystic fibrosis (CF) or chronic wounds, in which *S. aureus* and *P. aeruginosa* have been co-isolated, disease outcomes have been seen more severe compared to single species infections^1^. Due to the intrinsic mechanisms of adaptation, survival and resistance to multiple classes of antibiotics^9,10^, their strain- and environment-specific interactions could play an important role in disease progression, the antimicrobial therapy choice and the clinical outcome^11–13^. While interactions between *S. aureus* and *P. aeruginosa* have been widely reported during the course of chronic pulmonary diseases, such as CF and chronic obstructive pulmonary disease (COPD)^14^, little has been investigated regarding these interactions in acute LRTI or bronchial colonization. Transition from occasional colonization into persistent respiratory infections could be facilitated, among many other factors, by the ability of pathogenic bacteria to form biofilms and tolerate antibiotic treatments^13,15^. In addition, the properties of *S. aureus* and *P. aeruginosa* strains associated with acute LRTI differ from those that chronically colonize the airways of CF patients^16,17^. The complex network of interactions between them involve changes in microbial behavior^18,19^ which in turn could enhance adaptation mechanisms or pathogenicity of microorganisms in the respiratory tract, such as the mixed-species biofilm formation^20^.

Both pathogens utilize cell–cell communication via different quorum sensing (QS) systems to coordinate the expression of multiple virulence factors and resistance determinants upon environmental stress conditions, as well as to compete with other members of the microbial community^1,21^. In *S. aureus*, the *agr* (accessory gene regulator) system is the most well studied QS network contributing to virulence in model biofilm-associated infections, although its exact role varies according to the type of infection model used^22^. Surfactant peptides secreted by *S. aureus* in a QS-controlled fashion have been associated with biofilm detachment and consequent bacterial dissemination^23^. On the other hand, among the variety of QS signaling molecules in *P. aeruginosa*, quinolone-based signals, with 2-heptyl-3-hydroxy-4-quilonone (PQS) as the main effector molecule, seem to induce the production of virulence factors in the presence of Gram-positive organisms, such as *S. aureus* ^17,21,24^. Two other QS systems in *P. aeruginosa* (LasR and RhIR) work regulating PQS through its main effector molecule, N-acylhomoserine lactone (AHL)^1^.

The study of the microbial synergies or interferences between *S. aureus* and *P. aeruginosa* in the context of acute LRTI or bronchial colonization could help to establish the real clinical role of these microorganisms in polymicrobial infections, as well as to gain further insight on how both pathogens interact with each other in the respiratory tract.

In this work, we investigated co-cultures and mixed biofilms formed in vitro by clinical strains of *S. aureus* and *P. aeruginosa* co-isolated from clinical respiratory samples after careful assessment on clinical diagnosis. We also determined the influence of their exoproducts and cross-interference in their QS system activities in planktonic and biofilm cultures using an artificial sputum medium. Finally, the interaction between both species was further assessed in the *Galleria mellonella* acute infection model. Differential genotypes and virulence phenotypes were observed among the strains studied here, and yet common traits were still detected in the interaction between each coexisting pairs of *S. aureus* and *P. aeruginosa*. This work also highlights the importance of working with clinical strains over other studies using laboratory strains.

## Results

### Inter-strain phenotypic diversity among the *S. aureus* and *P. aeruginosa* co-isolated pairs

Five co-isolated pairs of *S. aureus* and *P. aeruginosa* were obtained from five patients admitted to the ICU from Hospital Universitari Germans Trias i Pujol that were classified as having tracheobronchitis (3) and bronchial colonization (2) (Table 1). The clinical strains were first characterized based on species-specific virulence phenotypes (Supplementary Tables S1 and S2). In addition, genetic classification of the isolated *S. aureus* strains was done by DNA microarray. The five *S. aureus* strains belong to four clonal complexes and those strains with the same multilocus sequence typing (MLST) profile display different virulence genes as determined by DNA microarray. The *agr* genotyping also revealed the phenotypic diversity of the strains, and Agr was inferred to be functional in the isolates SAR2746, SAR7115 and SAR5091 as determined through δ-haemolysin detention in CAMP assay. Strains SAR7244 and SAR10471 were non-haemolytic on blood agar.

**Table 1:** Identification of isolates and associated clinical information.

Isolate (ID)	Patient’s clinical data*						
	Sample isolation	Comorbidities	Respiratory study group†	ICU length of stay (days)	Tracheostomy	Days of MV	Development of respiratoryomplications
10471	Sputum	COPD/squamous cell lung carcinoma/lobectomy	BC	–	No	–	–
7244	ETA	Dyslipidemia	TB	18	No	18	Atelectasis/pneumothorax/respiratory failure
2746	ETA	AH/laryngeal neoplasia	TB	17	Yes	17	–
7115	ETA	AH/diabetes/encephalopathy	TB	64	Yes	34	Respiratory failure, exitus
5091	ETA	–	BC	18	No	5	–

*ETA: endotracheal aspirate; COPD: chronic obstructive pulmonary disease; AH: arterial hypertension; MV: mechanical ventilation.

†BC: bronchial colonization; TB: tracheobronchitis. Respiratory study groups according to the Clinical Pulmonary Infection Score (CPIS) as previously described^61^.

*P. aeruginosa* isolates also displayed significant diversity in terms of their genomic sequences, colony morphology and virulence related phenotypes. The MLST analysis shows that the five strains belong to different sequence types (STs) and the *P. aeruginosa* MLST database indicates a novel ST for strain PAR7244 (Table S2). Furthermore, none of the detected STs are part of the same clonal complex and none share more than two alleles in common. Virulence factors, such as swarming motility, fluorescent siderophore production, pyocyanin pigment and protease secretion were differentially expressed by all strains (Table S2 and Fig. S1).

### Clinical isolates of *P. aeruginosa* induce slow-growing colonies in *S. aureus*

**Figure 1:**
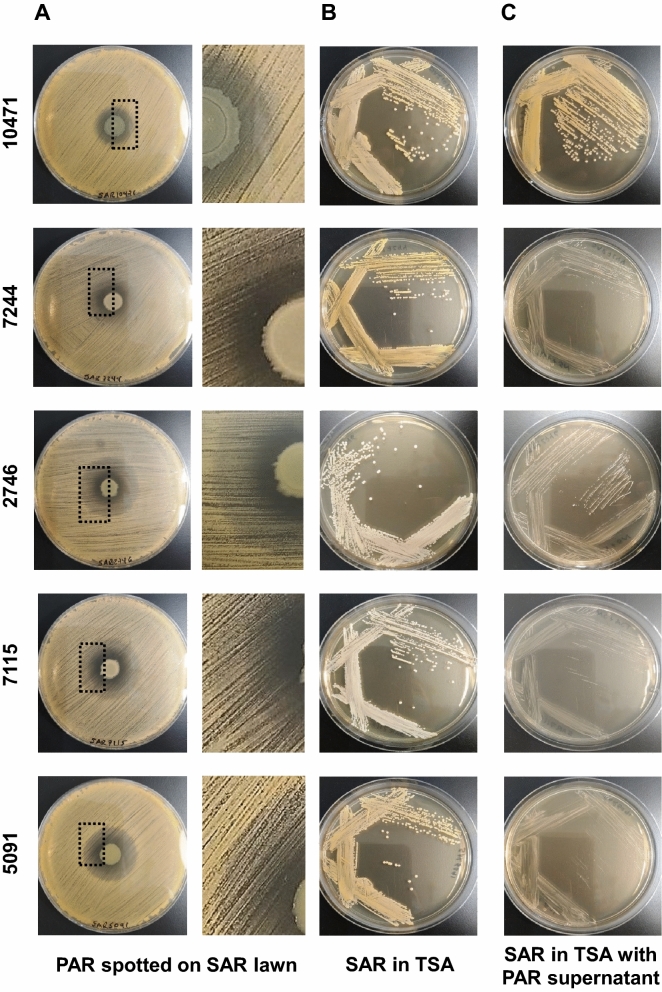
Interspecies growth competition assay on agar plates. (**A**) Colonies of *P. aeruginosa* (PAR) grown on a lawn of *S. aureus* (SAR) of the same co-isolated pair on tryptic soy agar (TSA) plates. Enlarged view of the boxed area is shown in the right panel. (**B** and **C**) *S. aureus* was streak-inoculated onto TSA plates with or without *P. aeruginosa* supernatant. Strain names are according to the origin of the sample (see Tables 1, S1 and S2).

To investigate how the *P. aeruginosa* and *S. aureus* co-isolated pairs from patients with tracheobronchitis or bronchial colonization coexist and interact with each other, we first studied growth competition by co-culturing the two organisms in agar plates. The presence of *P. aeruginosa* inhibited growth of *S. aureus* on TSA plates as indicated by the halo formed around the bacterial colonies (Fig. 1A). Inside the inhibition halos, total killing was only observed in a narrow zone surrounding the *P. aeruginosa* colony except for strain SAR10471; beyond this area *S. aureus* colonies grew slowly and lost pigmentation. This effect caused by the diffusion of an inhibitory substance in a solid medium, could indicate the secretion of antistaphylococcal molecules by *P. aeruginosa*. Besides, the presence of *P. aeruginosa* supernatant in the medium also induced the formation of these small non-pigmented colonies, except for the isolated pair with ID 10471 (Fig. 1B and C). *S. aureus* SAR10471 was less affected if considering the inhibition halo diameter and no slow-growing colonies were observed in the presence of PAR10471 supernatant. The observed phenotypic changes in the other four *S. aureus* strains upon contact with *P. aeruginosa* were transient. For these isolates, the inhibitory effect of *P. aeruginosa* on *S. aureus* growth could be related to the production of phenazine pigments such as pyocyanin according to the culture medium used (Fig. S1). When the strain PAR10471 was grown on TSB, no pigment production was detected. Interestingly, this strain is the one that showed the lowest proteolytic activity (Table S2).

### Pseudomonas aeruginosa affect growth and biofilm formation in S. aureus, but show synergism in the early stages of biofilm formation in artificial sputum medium

**Figure 2:**
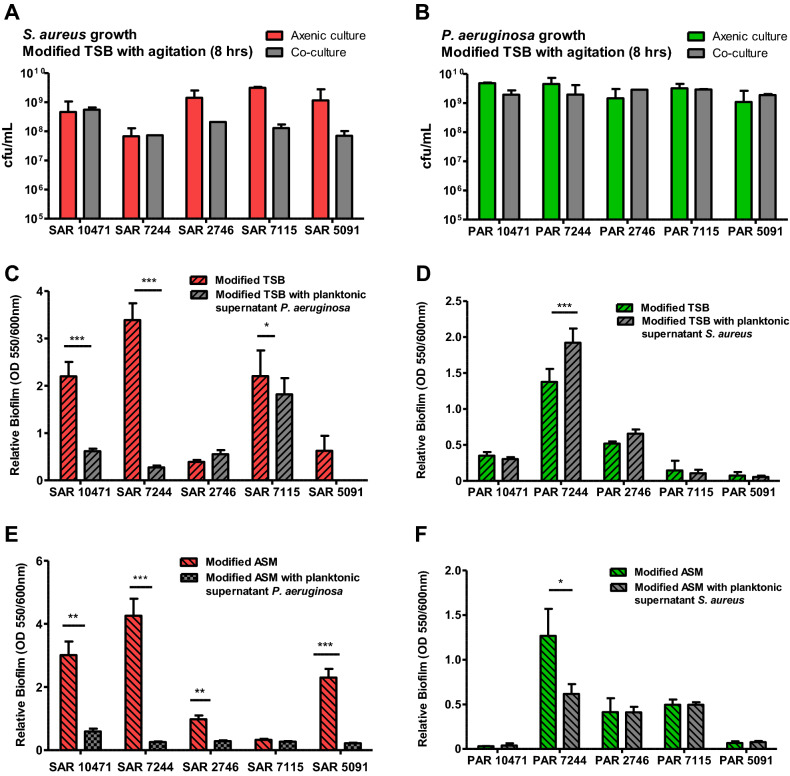
Effect of the interaction between clinical co-isolated pairs of *S. aureus* and *P. aeruginosa* on bacterial growth and biofilm formation. (**A** and **B**) Growth of *S. aureus* (SAR) and *P. aeruginosa* (PAR) strains in axenic culture and in co-culture with the corresponding co-isolated pair. The values represent the mean of viable bacterial count per ml (cfu/ml) after 8 h of cultivation at 37 °C with agitation. (**C** and **E**) Effect of the planktonic supernatants from *P. aeruginosa* on biofilm formation by the co-isolated *S. aureus* strain both in modified TSB or ASM medium at 37 °C. (**D** and **F**) Effect of the planktonic supernatants from *S. aureus* on biofilm formation by the co-isolated *P. aeruginosa* strain both in modified TSB or ASM medium at 37 °C. In all panels modified TSB consists of 0.5X TSB with 1% glucose, and modified ASM (artificial sputum medium) is a 1:1 mix of ASM (see methods) and 0.5X TSB with 1% glucose. When added, the planktonic supernatants are diluted 1:10 in culture medium. Error bars indicate standard deviation of biological replicates. *p < 0.05, **p < 0.01, ***p < 0.001 by Unpaired t test.

To study competition between *P. aeruginosa* and *S. aureus* during biofilm formation, we first evaluated growth interference in planktonic conditions. For this purpose the two species were inoculated simultaneously in 0.5X TSB with 1% glucose and allowed to grow until the end of the mid-logarithmic phase. The numbers of viable cells collected from each species in both mixed and axenic (control) cultures are shown in Fig. 2A and B. For all pairs, except for the co-isolated pairs identified as 10471 and 7244, the presence of *P. aeruginosa* inhibits the growth of *S. aureus* in mixed cultures with more than tenfold reductions in viable cells. It must be noted that the strains of *S. aureus* SAR10471 and SAR7244 are genetically related and belong to the same clonal complex. In general, the growth of *P. aeruginosa* was not significantly affected in mixed cultures. In the competition assays in TSA plates, strains SAR10471 and SAR7244 were the least affected by their corresponding *P. aeruginosa* isolates if we take into account the diameter of the inhibition halos (Fig. 1).

We also evaluated the effect of one species exoproducts on static biofilm formation on polystyrene plates by the other species using planktonic supernatants (Fig. 2C, D). Biofilm formation was tested in both 0.5X TSB supplemented with 1% glucose, a media that promotes robust biofilm growth for *S. aureus *in vitro^25^ and enhances their survival in the mixed biofilm^26^, and a TSB-based artificial sputum medium (ASM) to mimic growth in the cystic fibrosis lung habitat^27^. First, regarding the intrinsic capacity of each strain to form biofilm in both growth media (without the influence of the other species), a large phenotypic variability was observed between the strains of both species. For example, the *S. aureus* strains SAR2746 and SAR5091 with functional Agr form much less biofilm than the other ones. On the other hand, the *P. aeruginosa* strain PAR7244 is a strong biofilm former, while the strain PAR5091 almost does not form biofilm under the tested conditions. Despite this variability, and in almost all cases, the presence of *P. aeruginosa* supernatant significantly decreased biofilm formation by *S. aureus* under the two growth conditions used (Fig. 2C, E), with the exception of strain SAR2746 in TSB and strain SAR7115 in modified ASM*.* On the contrary, the presence of *S. aureus* supernatant did not affect biofilm formation by *P. aeruginosa*, except for the strain PAR7244 which showed variable results (Fig. 2D, F).

**Figure 3:**
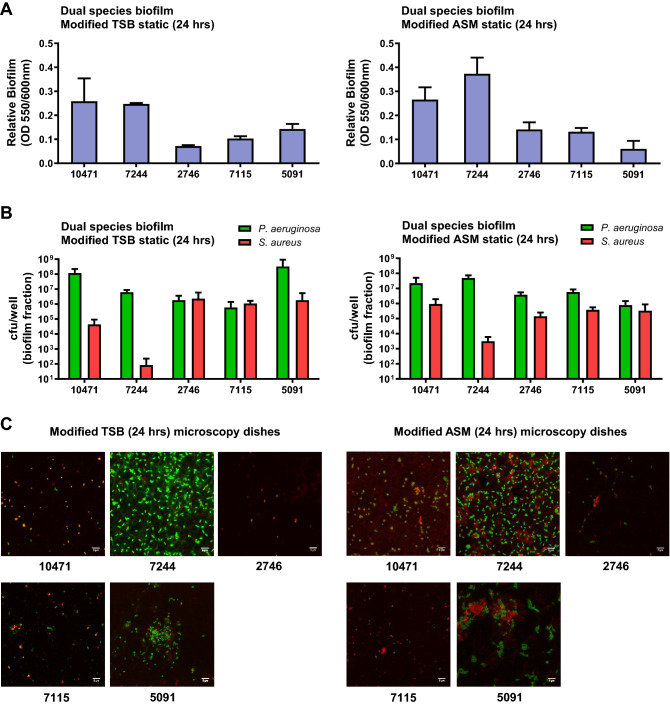
Attachment and microcolony formation in dual species static biofilms of clinical *S. aureus* and *P. aeruginosa*. Biofilm biomass measured by crystal violet method (**A**) and population of each bacterium in the mixed biofilm (**B**). Biofilms were grown under static conditions on polystyrene microtiter plates in 0.5X TSB supplemented with 1% glucose or in artificial sputum medium (ASM) mixed 1:1 with 0.5X TSB 1% glucose for 24 h. (**C**) Representative confocal laser scanning microscopy images of co-culture biofilms grown under static conditions on microscopy dishes for 24 h, and stained fluorescently for differentiation between species. Texas Red-X selectively binds to the surface of gram-positive bacteria to fluoresce red, while SYTOX counterstains the Gram negative fixed cells and fluoresces green. Scale bars represent 5 μm. In all experiments bacterial inoculation ratio was 10:1 (*S. aureus*:*P. aeruginosa*) and error bars indicate standard deviation of three biological replicates. Strain names are according to the origin of the sample (see Tables 1, S1 and S2).

We studied mixed biofilm on polystyrene plates after 24 h of static growth in both TSB-based media (Fig. 3). Both strains were inoculated simultaneously at a ratio of 10:1 (*S. aureus*: *P. aeruginosa*) to prevent *P. aeruginosa* from totally outcompeting *S. aureus*. Under these conditions, and after 24 h of growth, the total amount of biofilm formed was lower compared to single-species biofilm (Fig. 3A). Even so, both species were collected from the formed biofilms but in different proportions (Fig. 3B). Irrespective of the co-isolated pair and the growth medium, the ratio of the two species in the biofilms after 24 h cultivation was lower than in the initial inoculum, with a higher proportion of *P. aeruginosa*. Differential cfu counting showed that the ASM-based medium increases this ratio in favor of *S. aureus* in the pairs 10471, 7244 and 5091 (Fig. 3B). The dual species biofilm in both media under static conditions on microscopy dishes were also visualized by confocal microscopy and representative images are presented in Figs. 3C and S2. When grown as a single species biofilm, *S. aureus* formed extensive patches of biomass after 24 h of growth, except for strains SAR10471 and SAR7115 that grew forming microcolony structures in artificial mucus medium (Fig. S2). On the contrary, all *P. aeruginosa* strains appeared as microcolonies in both growth conditions after 24 h of growth, except for strain PAR7244 that formed uniform sheets of biomass. This is probably because TSB is less capable of supporting *P. aeruginosa* biofilm formation under static conditions^28^, and also because this species takes up to 3 days to develop mature biofilm in ASM^29,30^. During co-culture, attachment of *S. aureus* cells to the surface of plastic dishes was negatively affected and the strains merely formed some localized cell aggregates. The initial formation of these microcolonies was favored in the modified ASM medium (Fig. 3C). In co-culture in the sputum-like medium, *P. aeruginosa* cells appear to have clustered around cellular aggregates formed by *S. aureus*, promoting the initial stage of mixed biofilm formation.

### Exposure to *S. aureus* supernatant influences swarming motility in *P. aeruginosa*

**Figure 4:**
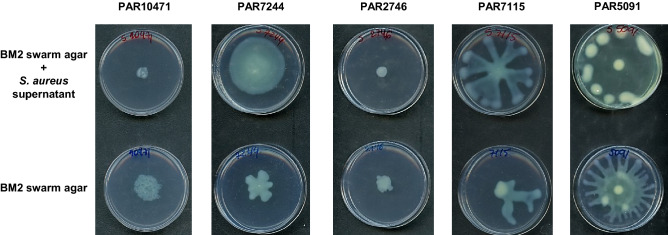
Effect of *S. aureus* supernatants on swarming motility of *P. aeruginosa*. Swarming motility was evaluated both in the absence and presence of the corresponding *S. aureus* supernatant diluted 1:10 in swarming medium (BM2 with 0.5% agar). Plates were incubated at 37 °C for 20 h. Images are representative of triplicate experiments.

The effect of the presence of *S. aureus* strain, tested as culture supernatant, on swarming motility of the *P. aeruginosa* co-isolated strain was evaluated and the results are summarized in Fig. 4. In the three *P. aeruginosa* strains PAR5091, PAR7115 and PAR7244 exhibiting a dendritic swarm pattern, swarming motility increased in the presence of cell-free supernatants from its *S. aureus* counterpart. On the contrary, *P. aeruginosa* PAR10471 strain showed reduction on swarming motility in the presence of supernatant of the *S. aureus* co-isolated pair. Strain PAR2746 did not show motility under any of the conditions tested.

### Species specific quorum-sensing activities are differentially regulated in co-cultures

**Figure 5:**
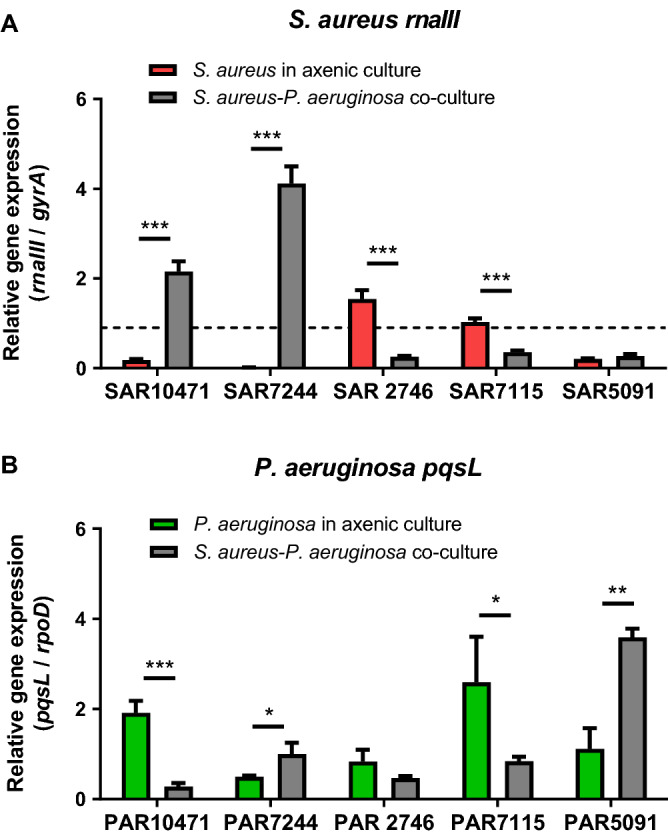
Differential deregulation of genes controlled by quorum sensing in co-culture. Relative expression of quorum sensing genes in co-cultures of *S. aureus* and *P. aeruginosa* in modified artificial sputum (ASM) medium (1:1 mix of ASM and 0.5X TSB with 1% glucose) assayed by qRT-PCR. (**A**) Effect of *P. aeruginosa* on *rnaIII* expression in *S. aureus*. Horizontal dashed line denotes the cut-off used to discriminate between Agr functional and dysfunctional strains (see methods). (**B**) Effect of *S. aureus* on *pqsL* expression in *P. aeruginosa*. In all experiments error bars indicate standard deviation of three biological replicates.

To evaluate the impact of the presence of one species on the quorum sensing activity of the other species of the co-isolated pair, we first studied the *agr* QS system of *S. aureus*. Expression of RNAIII, the effector of *agr* response, was measured by qRT-PCR in the five clinical strains grown under two conditions, in axenic cultures or in co-cultures with *P. aeruginosa*. ASM mixed 1:1 with 0.5X TSB supplemented with 1% glucose was used as the growth medium. When both species were grown together, the presence of *P. aeruginosa* affected the expression of the *rnaIII* gene compared with its expression in the axenic cultures in a strain-dependent manner (Fig. 5A). In the genetically related and Agr negative strains SAR10471 and SAR7244, *rnaIII* gene expression increased significantly (*P* < 0.001) in co-cultures. On the contrary, for the Agr positive strains SAR2746 and SAR7115 rnaIII expression decreased to levels that correlate with negative strains.

Using the same co-culture conditions in ASM modified medium, we studied the HQNO secretion system in *P. aeruginosa* strains by measuring expression of *pqsL* gene by qRT-PCR (Fig. 5B). PqsL is a mono-oxygenase that is required for the synthesis of the anti-staphylococcal compound HQNO which belongs to the PQS family^31^. In addition, expression of *pqsL* is suggested to play a role in PQS biosynthesis^32^. We had previously seen that the expression of *pqsL* is not detectable when the cells are grown in TSB medium; however, ASM medium positively regulated the expression of this gene in the *P. aeruginosa* strains. In co-culture with *S. aureus pqsL* expression was significantly affected, and once again it was in a strain dependent manner (Fig. 5B). The expression of *pqsL* significantly increased for the *P. aeruginosa* strains PAR5091 (*P* < 0.01) and PAR7244 (*P* < 0.05) in co-culture, the strains that showed the greatest negative impact on the growth of *S. aureus* (see Fig. 2). On the other hand, for the other three strains, the *pqsL* levels decreased in co-cultures.

The AHL quorum sensing system was also studied in *P. aeruginosa* isolates. All strains tested produced detectable levels of AHL, being the most producer strain PAR7115 and the strains PAR10471 and PAR2746 the lowest producer ones (Fig. S3). All strains synthesized long chain 3-oxo-AHLs including C8 and C10. In general, the presence of exoproducts from *S. aureus* did not significantly affect the production of AHL molecules by *P. aeruginosa* strains (Fig. S3).

### Virulence of *S. aureus* and *P. aeruginosa* strains alone or in co-infection in *G. mellonella* larvae

**Figure 6:**
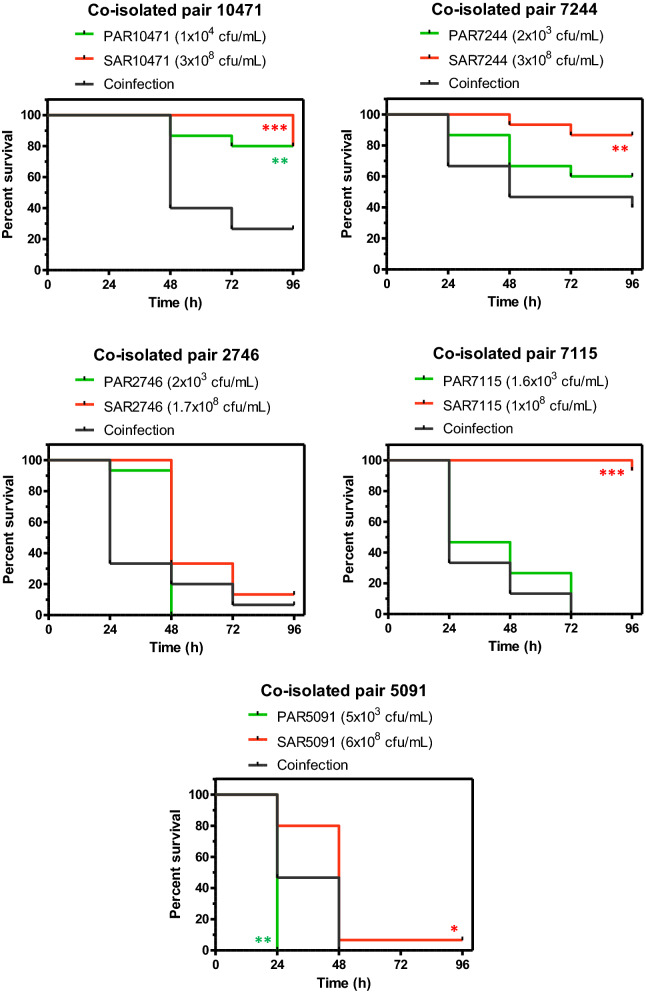
Co-infection in vivo shows an additive effect for the co-isolated pair with ID 10471. Survival of *Galleria mellonella* larvae over 96 h following infection with single species microorganisms (*P. aeruginosa* or *S. aureus*), or dual-species microorganisms (co-infection). Each panel represents a pair of co-isolated strains where each strain was used at the indicated infective dose prepared in PBS. Asterisks indicate significant differences relative to co-infected larvae, as assessed by the log-rank test. Only significant differences are shown.

Acute infection of *G. mellonella* larvae with individual clinical strains of *S. aureus* (1–6 × 10^6^ cfu/larva) resulted in larval survival between 80 and 95% at 72 h post-infection (Fig. 6) for strains 10471, 7115 and 7244. *S. aureus* strains 2746 and 5091 were more virulent with a survival rate of 13% and 7% respectively in the same time period. On the other hand, since *G. mellonella* is highly sensitive to *P. aeruginosa* injected into the hemolymph^33^, infections with only 15–100 cells per larva were done. Under these conditions, the *P. aeruginosa* strains 7115, 2746 and 5091 killed all larvae within 96 h (Fig. 6). However, mortality was lower with the strains 10471 and 7244. Co-infection experiments with *P. aeruginosa* and *S. aureus* co-isolated strains resulted in a significant decrease in survival (*P* < 0.01) only for the strain pair 10471 (Fig. 6). For the other co-isolated pairs, the mixed infection did not show significant differences in comparison to the corresponding single species inocula that showed the most virulence.

## Discussion

The interaction between clinical strains of *P. aeruginosa* and *S. aureus* has been extensively studied in recent years both in vivo and in vitro mostly from chronic infections^1,8,21,34–37^. Most of these studies have shown that when both bacteria are grown together, *P. aeruginosa* surpasses or decreases the *S. aureus* population. In the present work, this inhibitory effect of *P. aeruginosa* over its *S. aureus* counterpart has been observed for various clinical pairs co-isolated from the respiratory tract of patients with acute LRTI or bronchial colonization. However the strength of such an inhibitory effect has been strain specific. It has been shown that *P. aeruginosa* outcompetes *S. aureus* for the limited availability of required nutrients in the medium^38^. *P. aeruginosa* also produces an array of antimicrobial factors, which include respiratory inhibitors like hydrogen cyanide, pyocyanin, and the antistaphylococcal compound HQNO, as well as proteases that degrade wall components of *S. aureus*^21,39,40^. Particularly pyocyanin diverts electron flow and thus increases levels of products of oxygen reduction^41^. After lysis *S. aureus* could be an iron source for *P. aeruginosa* in cultures under iron-limiting conditions which contributes to the success of the latter^39^. Supplementary Fig. S4 summarizes the main interactions studied here between members of each co-isolated pair, and the phenotypic changes due to these interactions. Considering the high genetic variability of the clinical isolates and their clinical origin, it is not surprising that the interactions are strain-specific. Despite this, and the strong inhibitory effect exerted by *P. aeruginosa* on *S. aureus* under standard laboratory conditions, both species cooperate to survive in the mucosal environment of the respiratory tract.

The regulation of quorum sensing (QS) systems could play an important role in these dual species interactions. Secretion of anti-staphylococcal compounds is largely dependent on the modulation of the QS activity in *P. aeruginosa* in co-cultures. For example, HQNO is regulated by the quorum-sensing system PQS and it is involved in the increase of reactive oxygen species activity by disrupting the respiratory chain^1,42^. It has been shown that a *P. aeruginosa* mutant, deficient in the production of HQNO, does not efficiently kill *S. aureus*^19^. Exposure of *S. aureus* to HQNO has also been shown to down-regulate *agr* expression^20^. Beside this, several long-chain AHLs inhibit growth and virulence of *S. aureus* by reducing the *agr* activity^43^. All *P. aeruginosa* strains studied here synthesize long chain AHLs, and in general AHL production was not significantly affected by the presence of *S. aureus* exoproducts. However, co-culture with *S. aureus* seems to affect HQNO production in *P. aeruginosa* in a strain-specific manner. On the other hand, in *S. aureus* isolates with functional *agr* QS system, the exoproducts from co-isolated *P. aeruginosa* strains inhibit the expression of *rnaIII*, the regulatory effector molecule of this system. On the contrary, co-culture with *P. aeruginosa* activates *rnaIII* gene expression in strains with non-functional *agr* systems, which could also justify the specific strain pattern during the interaction between both species. QS network rewiring has been proposed as a strategy to coexist in the mucoid environment of the respiratory tract^44^. The observed increase in *agr* activity for strains SAR10471 and SAR7244 in co-cultures could correlate with a significant decrease in biofilm formation in the presence of *P. aeruginosa* supernatants (Fig. S4). Agr-mediated QS regulates many virulence factors in *S. aureus* that are important for the establishment of infection^45,46^. For instance, a functional Agr system is required for colonization in some infection models (e.g. epidermal), whereas reduced *agr* expression has been associated with increased adherence and biofilm production^46,47^.

Although this work is focused on strains isolated from patients with acute LRTI or bronchial colonization, the coexistence of *P. aeruginosa* and *S. aureus* can complicate both the course of the disease and the treatment decision making, promoting the transition into recalcitrant and chronic infections. For example, in CF patients carrying both microorganisms, co-infection is associated with worse disease outcomes and both pathogens seem to contribute additively to the disease severity^1,48^. Although as discussed before, *P. aeruginosa* impairs *S. aureus* growth, the co-occurrence of both microorganisms might also result in increased expression of some staphylococcal virulence factors^49^. Furthermore, *S. aureus* can survive in the presence of *P. aeruginosa* by the selection of tiny, non-pigmented colonies known as small-colony variants (SCVs). This phenotype is typically transient and it appears upon contact with exoproducts secreted by *P. aeruginosa*, such as pyocyanin and HQNO, that block the respiratory chain of *S. aureus*^40,50,51^. This phenotype can also be irreversible due to permanent genetic changes. *S. aureus* is able to persist in the airways of patients with CF by transforming into SCVs^40^, and this phenotype has been observed in biofilms after antibiotic therapies^52^. We have shown formation of slow-growing colonies of *S. aureus* by co-culturing on agar plates with *P. aeruginosa*, as demonstrated by others using CF patient co-isolates^53^. Regarding dual species biofilm formation, it has been shown that if *S. aureus* cells are able to survive killing by *P. aeruginosa*, certain extracellular factors of *P. aeruginosa* protect them within the biofilm matrix^54,55^. Interactions between *S. aureus* and *P. aeruginosa* could be reciprocally beneficial to each organism in terms of initial colonization and pathogenicity where co-aggregation plays a crucial role^56^. Here we also show that in co-culture, *S. aureus* cells start to attach and co-aggregate with *P. aeruginosa* on the plastic surface despite the strong inhibitory effect exerted by the latter. This synergistic effect is favored in conditions mimicking the CF pulmonary mucus.

On the other hand, the presence of molecules secreted by *S. aureus* increases *P. aeruginosa* virulence by enhancing the production of extracellular virulence factors such rhamnolipids, pyocyanin, elastase and quinolone-based signals^1^. A more recent study showed that *S. aureus* produces proteinaceous factors responsible for promoting *P. aeruginosa* surface motility^57^. In the present study exposure to *S. aureus* supernatant influenced swarming motility in the *P. aeruginosa* co-isolated strain, however this did not affect its ability to form biofilm. It has also been found by others that *P. aeruginosa* isolates from co-infected patients are less competitive with *S. aureus*^36^. Additionally, the presence of N-acetyl glucosamine from Gram-positive bacteria has been shown in vivo to increase the virulence of pathogenic *P. aeruginosa* in a *G. mellonella* infection model^58^. We have also detected this additive effect in the acute infection model in *G. mellonella*, but only for the co-isolated pair which showed the smallest competitive effect of *P. aeruginosa* on *S. aureus* growth (Fig. S4). Competition between *S. aureus* and *P. aeruginosa* has been also shown by others in the mouse model of acute lung infection^53,59^. In these studies it was also demonstrated that *S. aureus* profits from the presence of *P. aeruginosa* and the interactions between both species could be strain specific.

The processes and interactions governing biofilm development are directly involved in the pathogenesis of polymicrobial infections^60^. The functional and structural organization of both microorganisms in biofilms is complex and it is mainly regulated by their QS systems^2^. Within the biofilm, cells can be transformed phenotypically to become more resistant to treatments promoting the chronicity of infections. Dual species interactions within biofilms are dependent on both *S. aureus* and *P. aeruginosa* strains^1,56^, and this might explain the inconsistency between each co-isolated pair through all the experiments. Significant phenotypic differences were observed among the co-isolates studied here including differences in traits important for successful lung colonization. Considering the origin of the strains and their phenotypic diversity, our results add more clues on the complex interactions that take place between both species in the respiratory tract during acute LRTI or simply during a temporary colonization. Although strain-specific, these interactions could play an important role in disease progression and the clinical outcome in LRTI. This study also suggests the importance of the QS in dual species biofilm-related infections, making interference on these systems a promising therapeutic option. To deepen this knowledge, further studies should be conducted, especially how global gene expression and metabolome are affected in co-cultures.

## Methods

### Clinical isolates

Ten clinical isolates were included in the study. *S. aureus* and *P. aeruginosa* strains included were isolated simultaneously in the same respiratory sample (4 endotracheal aspirates and one sputum sample) (co-isolated) from five patients admitted to the ICU from Hospital Universitari Germans Trias i Pujol, a tertiary care hospital in Badalona (Spain) (Table 1). The patients were classified into respiratory study groups according to the Clinical Pulmonary Infection Score (CPIS) and as previously described^61^. At inclusion, all patients except one were under mechanical ventilation. Ethical approval was provided and the need for informed consent was waived by the Institutional Review Board: Comitè d’Ètica de la Investigació de l’Hospital Germans Trias i Pujol. Patient consent was waived because material used were strains and clinical data were anonymized. Sample collection followed the standard of care and was based on suspicion of infection.

Frozen glycerol stocks (15%) of the isolates were stored at -80˚C until use. For some experiments, the reference strains *P. aeruginosa* PAO1 and *S. aureus* RN6390B were included as controls. Unless otherwise stated, cultures were routinely grown in tryptic soy broth (TSB; BD Difco, Le Pont de Claire, France) at 37 °C and maintained on LB agar plates.

### Mono-culture and co-culture planktonic growth

All growth curves experiments were done in 0.5X TSB supplemented with 1% glucose at 37 °C with agitation on a rotary shaker at 200 rpm. Overnight individual cultures of *S. aureus* and *P. aeruginosa* strains were diluted to an OD_550_ of 1.0 and used to inoculate (1:20) axenic or mixed cultures. For co-culture growth curves, bacteria were inoculated simultaneously at an equal ratio (1:1). Samples were collected every hour, serial diluted in PBS, and then seeded onto the selective media MacConkey agar (BD Difco) for *P. aeruginosa* and Mannitol salt agar (Conda, Madrid, Spain) for *S. aureus*. The agar plates were incubated at 37 °C for 24 h and the number of viable cells was determined.

### Preparation of cell-free culture supernatant

Overnight cultures for each individual strain of both species were prepared in 100 mL of 0.5X TSB supplemented with 1% glucose for 18 h at 37 °C with shaking at 200 rpm. Planktonic supernatants were obtained after centrifugation at 4000 g for 20 min at 4 °C, followed by sterile filtration using a 0.22 µm-pore-size filter (Merck Millipore, Germany) and storage at − 20 °C until use. Before the use, supernatants were adjusted to pH 7.0, supplemented with 0.4% glucose and oxygenated by mixing.

### Growth competition assay on agar plates

To prepare a bacterial lawn, overnight cultures of *S. aureus* were adjusted to OD_550_ of 0.01 and spread onto tryptic soy agar (TSA) plates using a sterile cotton swab. Five microliters of logarithmic cultures of *P. aeruginosa* isolates were spotted on the competitor lawns using a micropipette. In addition, fresh colonies of *S. aureus* were picked and streak-inoculated onto TSA plates with or without 10% (v/v) culture supernatant of *P. aeruginosa* grown in TSB. Plates were incubated 24 h at 37 °C.

### Haemolysin activity in *S. aureus*

Hemolytic activities and Agr functionality of *S. aureus* were inferred using the classical CAMP synergistic haemolysis test on blood agar plates^62^. In CAMP assay, strains were tested against *S. aureus* strain RN4220 which produces only β-haemolysin. After incubation at 37 °C for 24 h, the plates were refrigerated to increase β-hemolytic activity.

### DNA microarray-based genotyping of *S. aureus*

Genotypic characterization of *S. aureus* clinical isolates was performed with a species-specific genotyping DNA microarray (Alere Technologies GmbH, Jena, Germany), which detects specific resistance and virulence genes and their allelic variants and allows the assignment of isolates to MLST clonal complexes. PCR amplification and hybridization were performed following the manufacturer’s instructions. An image of the array was recorded and analyzed using a designated reader and software (Arraymate, Iconoclust, Alere Technologies)^63^.

### MLST typing of *P. aeruginosa* isolates

For each isolate, DNA was extracted using the DNeasy Ultra Clean Microbial kit (Qiagen). Nextera XT kit (Illumina Inc., San Diego, United States) was used for dual-indexed library preparation and paired-end sequencing was performed using the Illumina MiSeq platform (Illumina Inc., San Diego, United States) at the Germans Trias i Pujol Research Institute. The whole DNA content of five strains was sequenced with the Illumina technology. Contig sequences were uploaded to the *Pseudomonas* MLST database (https://pubmlst.org/organisms/pseudomonas-aeruginosa) to define the sequence types (STs) according to the scheme previously proposed for *P. aeruginosa*^64^.

### Colony morphology and virulence phenotypes in *P. aeruginosa*

Pigment production and colony morphology was assessed by growing strains on King A medium (Conda) at 37 °C for 24 h followed by visual inspection. The fluorescence of colonies was also detected under UV light using a table transilluminator. Swarming motility was assayed by point inoculation on BM2 swarm agar plates containing 0.5% (w/v) agar as previously described^65^. Qualitative determination of proteolytic activity was performed on skimmed milk agar at a final concentration of 1.5% skim milk. Tested strains were spotted on protease plates and incubated 24 h at 37 °C, the formation of a clear zone around the colony was considered as protease positive.

### Biofilm formation on polystyrene microtiter plates

Biofilm formation was determined in 96-well polystyrene microtiter plates (BRANDplates, no. 781662, Germany) with a staining method using crystal violet^25,66^. All biofilms were obtained using 0.5X TSB supplemented with 1% glucose or an artificial sputum medium (ASM)^27^ mixed 1:1 with 0.5X TSB 1% glucose. Briefly, overnight cultures of individual strains grown in LB broth were diluted in the culture media used for biofilm assay to an OD_550_ of 0.01 for *P. aeruginosa* and 0.05 for *S. aureus*. The diluted cultures were transferred to wells of polystyrene microtiter plates (200 µl per well) and incubated at 37 °C for 24 h under an aerobic atmosphere without agitation (static biofilm). After incubation, the OD_600_ of each well was measured using a multiwell plate reader to measure planktonic growth in each well. The wells were then gently washed three times with distilled water, dried for 60 min at 60 °C, and stained with 0.1% (w/v) crystal violet for 15 min. Then, the plate was rinsed with distilled water, dried at 37 °C and the crystal violet was extracted with 30% (v/v) acetic acid. The amount of biofilm was quantified by measuring the OD_550_ of dissolved CV using the plate reader. Biofilm formation was normalized to cell growth (OD_620_) to calculate relative biofilm formation. Each reported value corresponds to the arithmetic mean and standard deviation of two biological replicates, each with 8 to 12 replicates per strain and condition. To evaluate the effect of one species supernatant on biofilm formation by the other species of the co-isolated pair, 10% planktonic supernatant was added to growth media and the assay was performed as previously described. For co-culture biofilm, overnight cultures of clinical strains were diluted to an OD550 0.01 for *P. aeruginosa* and 0.1 for *S. aureus* and mixed (1:1, v/v). Each well was inoculated with 200 µl of each bacterial suspension and incubated at 37 °C for 24 h. To enumerate viable bacteria in the biofilms, after removal of planktonic culture, and after several washes of the plates with PBS, the biofilm fraction was removed by treatment with 250 µl of 0.1% Triton X-100 in PBS. Biofilm bacteria were then scraped and vortexed in the plate for 2 min and biofilm fractions were then serially diluted and plated in selective media. Additional non-treated wells were stained with crystal violet for total biomass quantification.

### Confocal laser scanning microscopy (CLSM) of *S. aureus* and *P. aeruginosa* biofilms

Single species and co-culture biofilms of *S. aureus* and *P. aeruginosa* were grown in microscopy-quality 35 mm sterile plastic dishes (µ-Dish ibidi, no. 81156, Germany). Biofilms were grown using 0.5X TSB supplemented with 1% glucose or ASM mixed 1:1 with 0.5X TSB 1% glucose. For single species biofilm cultivation, overnight cultures of clinical strains grown in TSB were diluted in both biofilm media to an OD_550_ of 0.01 and 0.05 for *P. aeruginosa* and *S. aureus* respectively. For co-culture biofilm, overnight cultures of clinical strains were diluted to an OD_550_ 0.01 for *P. aeruginosa* and 0.1 for *S. aureus* and mixed (1:1, v/v). Each dish was inoculated with 1 mL of each bacterial suspension and incubated at 37 °C for 24 h. Then, the dishes were gently washed with distilled water, allowed to air dry for several minutes in inverted position and 1 mL of 4% paraformaldehyde in PBS (0.1 M) was added. After incubation for 15 min, the dishes were gently washed twice with distilled water. Bacterial staining was made using the reagents SYTOX Green and Texas Red-X-WGA (ViaGram Red^+^ Bacterial Gram Stain and Viability Kit V-7023; Molecular Probes) for general nucleic acid and Gram-positive specific staining, respectively. Cells with damaged membranes stain fluorescent green with SYTOX Green. Texas Red-X-WGA was diluted 1:50 and SYTOX Green was diluted 1:500, and incubation was for 30 min. Images were recorded on an inverted Leica TCS-SP5 confocal microscope using an × 63/1.4 objective (Servei Microscòpia, UAB, Barcelona). The wavelengths of the excitation and emission lasers were, respectively: green channel (480/500 nm) to SYTOX Green and red channel (490/635 nm) to Texas Red-X-WGA. 3D images were obtained using the Imaris software package (V 6.1; Bitplane). The experiments were carried out in duplicate and at least two sections were acquired with an interval between sections of 0.17 μm.

### Reverse transcription and quantitative RT-PCR

RNA was extracted from single species cultures or co-cultures using the RNeasy Mini Kit (Qiagen, USA) according to the manufacturer’s instructions. All experiments were done in 10 mL of ASM mixed 1:1 with 0.5X TSB 1% glucose at 37 °C with agitation. Culture inoculation was performed in the same way as for CLSM. Samples were collected after 24 h of growth and were adjusted to an OD_550_ of 1.0, 0.5 mL of bacterial cultures were treated with two volumes of RNAprotect (Qiagen, USA), incubated for 5 min at room temperature and centrifuged at 5000 g. The pellet was resuspended in Tris–EDTA buffer containing 400 μg/ml lysozyme (Sigma-Aldrich, USA) for *P. aeruginosa* and/or 200 μg/mL of lysostaphin (Ambion, USA) for *S. aureus* and incubated for 30 min at 37˚C, followed by proteinase K treatment for 20 min under agitation. RNA was quantified using NanoDrop ND-1000 spectrophotometer (Thermo Fischer, USA) and the absence of DNA was verified by PCR. Reverse transcription was performed using Maxima First Strand cDNA Synthesis system (Thermo Fischer, USA) according to manufacturer’s instructions. For detection and quantification of *agr* functionality in *S. aureus*, the quantitative RT-PCR was done as previously described^67^ with modifications. Amplification of *rnaIII* gene was done with primers rnaIIIFW, 5’-GAAGGAGTGATTTCAATGGCACAAG-3’, and rnaIIIRV, 5’ GAAAGTAATTAATTATTCATCTTATTTTTTAGTGAATTTG-3’. For *P. aeruginosa* isolates the expression of *pqsL* gene was used to examine the PQS system as previously described^36^. The primers used for the analysis of the *pqsL* gene expression were: pqsLFW, 5’- CGGTATCGCCTCCTACGTG-3′, and pqsLRV 5′-GGAAGCTCACCACCAGTCG-3′. The amplification products were detected using the SsoAdvanced universal SYBR Green supermix (Bio-Rad, USA). Results from experiments performed in triplicate were normalized to the expression of species-specific reference genes. Gyrase *gyrB* and RNA polymerase sigma factor *rpoD* genes were used as the normalizing genes for *S. aureus* and *P. aeruginosa* respectively using the following oligonucleotide pairs: gyrFW, 5′-CCAGGTAAATTAGCCGATTGC-3′ and gyrRV, 5′-AAATCGCCTGCGTTCTAGAG-3′; and rpoFW, 5′-CGATCGGTGACGACGAAGAT-3′ and rpoRV, 5′-GTCACATCGAACTGCTTGCC-3’. *S. aureus* RN6390B was used as a positive control for *rnaIII* expression. We defined a strain to be Agr functional if *rnaIII* expression levels were at least tenfold lower than the positive control.

### Detection of AHLs production

AHLs were detected in bioassays by determining β-galactosidase activity using a sensor strain of *Agrobacterium tumefaciens* KYC55 carrying the plasmids pJZ372, pJZ384 and pJZ410, as previously described^68,69^ with few modifications. Briefly, *A*. *tumefaciens* KYC55 strain was grown in 20 mL of BM2 medium at 30 °C for 18 h. Cells were harvested and reconstituted with 10 mL of fresh BM2 and added to 40 mL of temperate BM2 medium containing 2% of Noble Agar (BD Difco) and 80 µg/mL of X-Gal. AHLs produced by *P. aeruginosa* strains were extracted with acidified ethyl acetate from cell-free culture supernatants as previously described^70^ and were evaluated both in bioassay plates and by thin layer chromatography (TLC) coupled to bioassay^70^. Presence of blue halos or spots indicates AHL production. *P. aeruginosa* PAO1 was used as a positive control of AHLs production.

To assess the effect of exoproducts of *S. aureus* on the AHL synthesis by *P. aeruginosa*, overnight cultures of *P. aeruginosa* strains grown in LB were diluted in fresh medium to an OD_550_ of 1.0. The AHL bioassay plates were prepared with and without the addition of 1:10 diluted supernatant of the corresponding *S. aureus* co-isolate and were seeded with 2 µL of the diluted culture and incubated at 30 °C for 24 h. The diameters of the blue halos were compared between both conditions.

### Infection of *Galleria mellonella* larvae

Larvae of *Galleria mellonella* were obtained from our own hatchery and only healthy-looking larvae weighing 200–300 mg and showing no signs of melanization were used in the experiments. To prepare bacterial inocula, *P. aeruginosa* and *S. aureus* isolates were grown overnight at 37°C in 10 mL of LB and 0.5X TSB supplemented with 1% glucose, respectively. Then, cells from individual cultures were sedimented by centrifugation at 5000 g, washed in PBS and adjusted to contain approximately the desired dose. This inoculum size was selected based on pilot studies that have determined the optimal infective dose of each strain required to kill *G. mellonella* over a 24–96 h period. The selected doses ranged from 10^1^ to 10^2^ cfu per larvae for *P. aeruginosa* and from 10^6^ to 4 × 10^7^ cfu per larvae for *S. aureus* isolates. Larvae were infected with *P. aeruginosa* or *S. aureus* or co-infected with *P. aeruginosa* and *S aureus* of the same co-isolated pair. Mixed infected larvae were inoculated with the same bacterial burden of *P. aeruginosa* and *S aureus* as those inoculated with single species microorganisms. Fifteen larvae per group were infected through the left proleg with 10 μL of each bacterial suspension using a 50 μl Hamilton Microliter syringe and incubated in the dark at 30 °C in empty petri dishes. Bacterial burden of the doses was confirmed by plating on LB agar medium. Mortality was determined every 24 h. Larvae were considered dead when they no longer responded to touch and turned black which correlates with total melanization.

### Statistical analysis

All statistical analyses were performed by GraphPad Prim software (ver. 5.0; GraphPad Inc, San Diego, USA), considering as statistically significant a *p*-value less than 0.05. For infection experiments Kaplan–Meier survival curves were plotted using GraphPad Prism and statistical significance was determined using the log-rank (Mantel-Cox) test.

### Statement

Experiments were performed in accordance with relevant guidelines and regulations.

## Supplementary Information

Supplementary Information.

## Data Availability

Whole Genome Shotgun projects for sequences identified in this study have been deposited at DDBJ/ENA/GenBank (accession codes are listed in Table S2). MLST information is available at the *Pseudomonas* MLST database.
