# Supplementary material for: Strain-specific interspecies interactions between co-isolated pairs of Staphylococcus aureus and Pseudomonas aeruginosa from patients with tracheobronchitis or bronchial colonization
Source: Sci Rep. 2022 Mar 1;12:3374. doi: 10.1038/s41598-022-07018-5 (PMC8888623; doi:10.1038/s41598-022-07018-5)
Supplement: Supplementary file 1 — Supplementary Information. [file 41598_2022_7018_MOESM1_ESM.pdf]

SUPPLEMENTARY INFORMATION

Strain-specific interspecies interactions between co-isolated pairs of *Staphylococcus aureus* and *Pseudomonas aeruginosa* from patients with tracheobronchitis or bronchial colonization

Meissiner Gomes-Fernandes,[1,2,3] Andromeda-Celeste Gomez,[2,4] Marc Bravo,[2,4] Pol Huedo,[2,4] Xavier Coves,[2,4] Cristina Prat-Aymerich,[1,5,6] Isidre Gibert,[2,4] Alicia Lacoma,[1,5]* and Daniel Yero[2,4]*

[1]Microbiology Department, Hospital Universitari Germans Trias i Pujol, Institut d'Investigació en Ciències de la Salut Germans Trias i Pujol, Universitat Autònoma de Barcelona (UAB), Badalona, Spain;

[2]Institut de Biotecnologia i de Biomedicina (IBB), UAB, Barcelona, Spain;

[3]CAPES Foundation, Ministry of Education of Brazil, Brasília, Brazil;

[4]Departament de Genètica i de Microbiologia, UAB, Barcelona, Spain;

[5]CIBER Enfermedades Respiratorias, CIBER, Instituto de Salud Carlos III, Badalona, Spain;

[6]Julius Center for Health Sciences and Primary Care, University Medical Center Utrecht, Utrecht University, The Netherlands.

These authors contributed equally: Meissiner Gomes-Fernandes and Andromeda-Celeste Gomez.

*Corresponding Authors: Alicia Lacoma, alacoma@igtp.cat and Daniel Yero daniel.yero@uab.cat.

This document includes:

Supplementary Tables S1 – S2

Supplementary Figures S1 – S4

Supplementary information

Table S1. Summary of genotypic and phenotypic characteristics of *S. aureus* clinical strains co-isolated with *P. aeruginosa*. Strain name matches to sample ID from which co-isolated pairs were obtained.

Strain ID	Molecular characterization using the StaphyType DNA microarray[a]						Haemolysin [b]
	MLST CC	*agr* allele	Presence of virulence genes				
			sak	*chp*	*scn*	*tst1*	
SAR10471	30	III	+	+	+	+	none
SAR7244	30	III	-	-	-	+	none
SAR2746	398	I	-	+	+	-	delta, alpha
SAR7115	121	IV/I	+	-	+	-	delta, alpha, beta
SAR5091	5	II	+	+	+	-	delta, alpha

[a] MLST Clonal Complex (CC). Accessory gene regulator (agr), staphylokinase (sak), chemotaxis inhibitory protein (chp), staphylococcal complement inhibitor (scn) and the toxic shock syndrome toxin 1 gene (tst1). Two *agr* allele types indicates ambiguous results.

[b] Haemolysin activity determined through CAMP assay (see supplementary figure S1).

Table S2. Summary of genotypic and phenotypic characteristics of *P. aeruginosa* clinical strains co-isolated with *S. aureus*. Strain name matches to sample ID from which co-isolated pairs were obtained.

Strain ID	MLST ST[a]	Colony morphology[b]			Proteolytic activity (halo diameter in mm)[c]
		Mucoidity	Pigments in King's agar medium	Swarming motility	
PAR10471	299	non-mucoid	deep bluish green	fractal-like	12.3 ± 1.2
PAR7244	3873	non-mucoid	light bluish green	dendritic	18.7 ± 0.6
PAR2746	253	non-mucoid	light bluish green	non-swarming	18.7 ± 1.2
PAR7115	3695	non-mucoid	light bluish green	dendritic	15.3 ± 1.5
PAR5091	446	non-mucoid	pale green (intense fluorescence)	dendritic	16.7 ± 0.3

[a] MLST sequence type (ST) are assigned by the PubMLST *P. aeruginosa* typing database (https://pubmlst.org/Pseudomonasaeruginosa/) based on whole genome sequencing. Whole Genome Shotgun projects has been deposited at DDBJ/ENA/GenBank under the accession JAKLAX000000000 (PAR5091), JAKLAY000000000 (PAR7115), JAKLAZ000000000 (PAR2746), JAKLBA000000000 (PAR7244) and JAKLBB000000000 (PAR10471).

[b] Pigment production and swarming motility are shown in Figures S1 and 4 respectively.

[c] Determination of proteolytic activity on skim milk agar plate.

Supplementary Figure S1

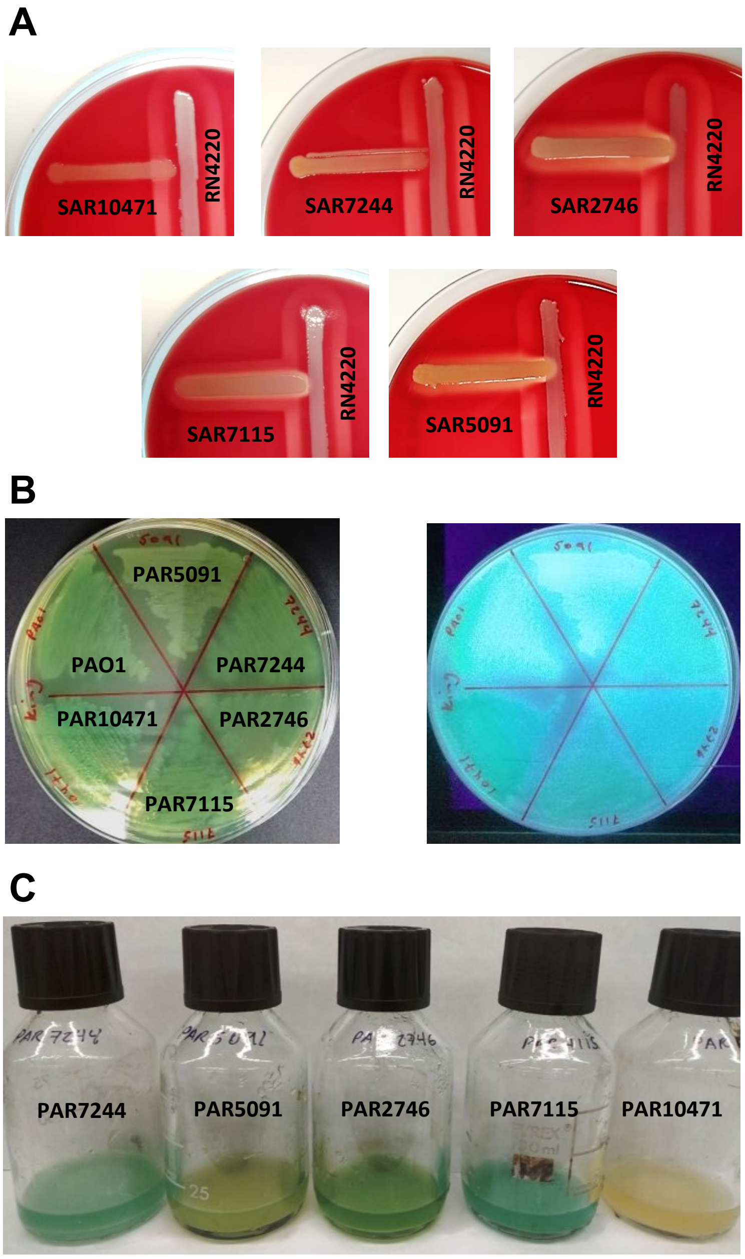

Figure S1. Phenotypes shown by the strains under study of *S. aureus* (SAR) and *P. aeruginosa* (PAR) under different growth conditions. (A) Haemolytic activities of SAR clinical strains determined by cross-streaking perpendicularly to a β-haemolysin producer strain (*S. aureus* RN4220) on a sheep blood agar plate. Pigment production by PAR strains in King A medium (B) and TSB (C). The fluorescence of colonies grown in King A medium was detected under UV light (right panel in B). *P. aeruginosa* PAO1 was included as a control for the production of pyocyanin and fluorescent pigments. The blue-green color in TSB cultures (C) changes with aeration, which indicates the presence of phenazine pigments.

Supplementary Figure S2

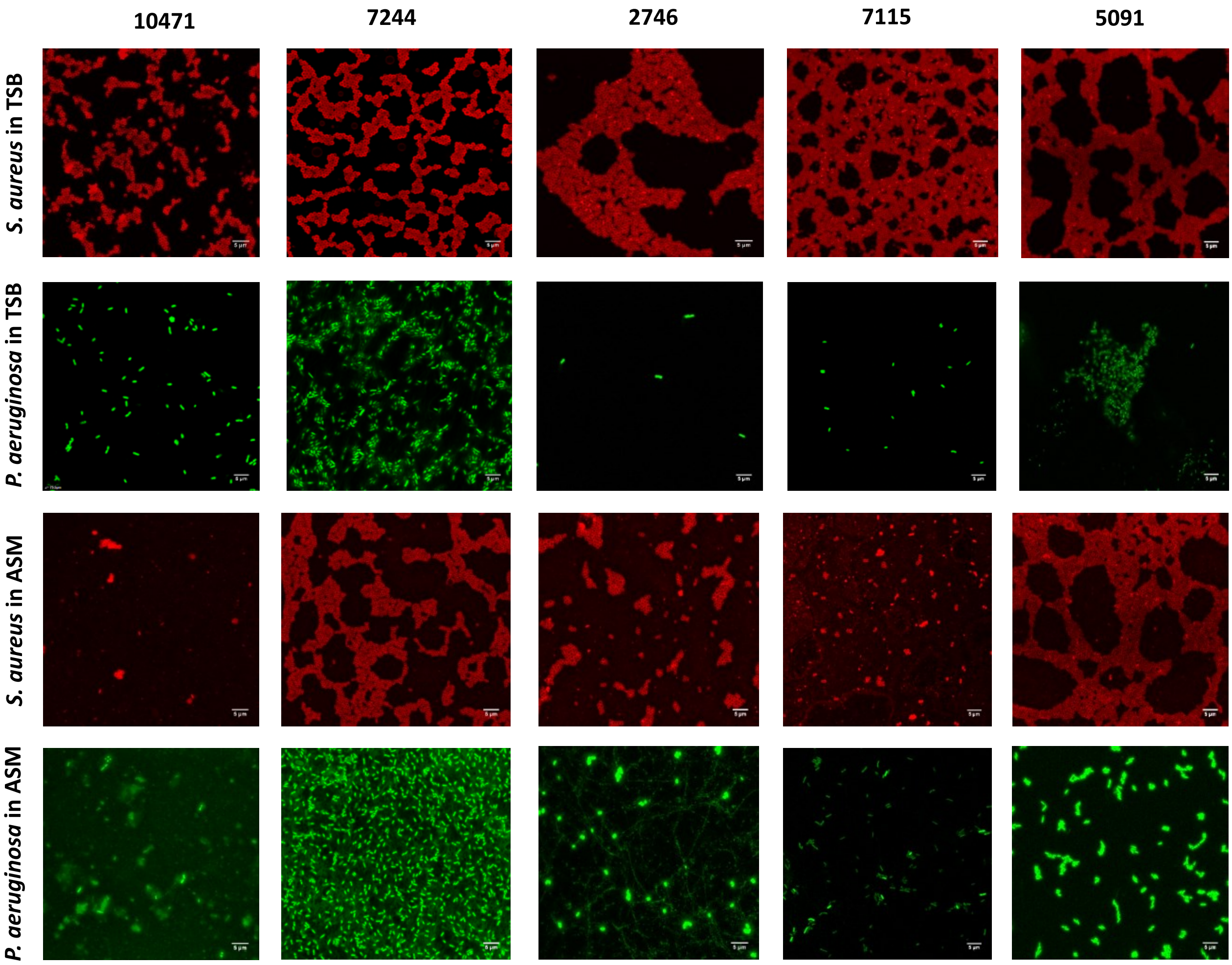

Figure S2. Representative confocal laser scanning microscopy images of single species biofilms of *S. aureus* and *P. aeruginosa* clinical co-isolates. Biofilms were grown under static conditions on microscopy dishes in 0.5X TSB supplemented with 1% glucose or in artificial sputum medium (ASM) mixed 1:1 with 0.5X TSB 1% glucose for 24 hours, and stained fluorescently. Texas Red-X selectively binds to the surface of gram-positive bacteria to fluoresce red, while SYTOX counterstains the Gram negative fixed cells and fluoresces green. Scale bars represent 5 µm.

Supplementary Figure S3

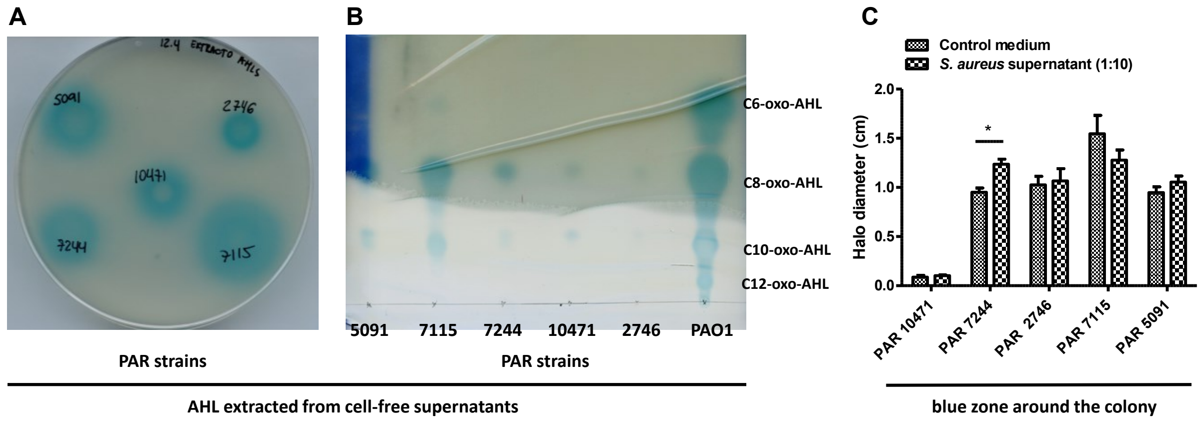

Figure S3. N-acylhomoserine lactone (AHLs) produced by the *P. aeruginosa* (PAR) clinical strains. AHLs production was determined by the presence of zones of blue pigmentation (hydrolysis of X-Gal) produced by the *Agrobacterium tumefaciens* KYC55 (pJZ372, pJZ384 and pJZ410) sensor. AHLs extracted from culture supernatants of each PAR strain were spotted (5 μL) on bioassay plate (A) or separated by TLC and visualized by means of the indicator strain in a bioassay (B). Effect of *S. aureus* supernatant on AHL production by *P. aeruginosa* strains (C). Diluted bacterial cultures were directly spotted on bioassay plates with or without supernatant added.

Supplementary Figure S4

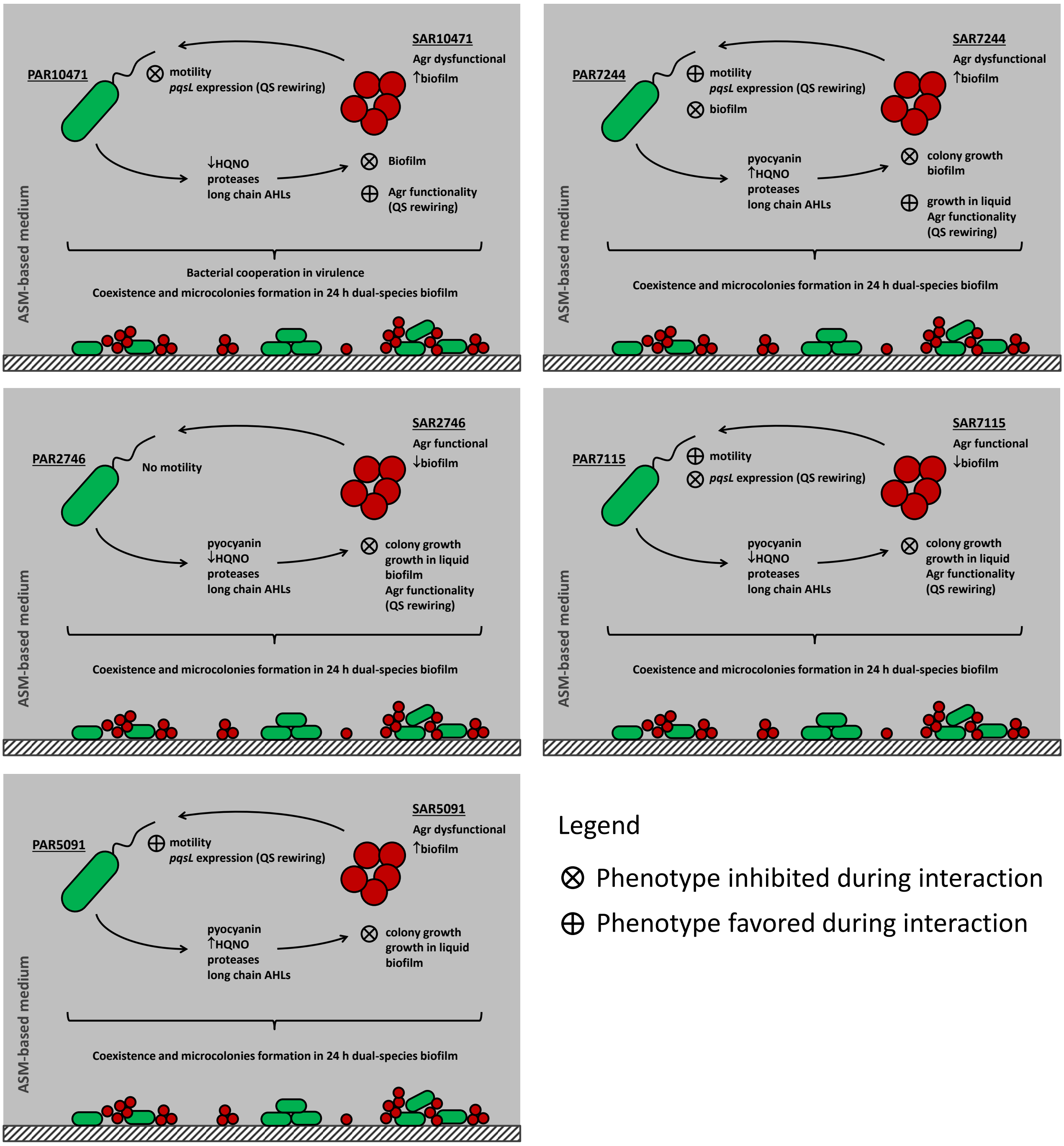

Figure S4. Schematic representation of the interactions studied between each of the co-isolated pairs of *P. aeruginosa* (PAR) and *S. aureus* (SAR). Each panel summarizes the phenotypes affected during the interaction and the global effect on biofilm formation in modified ASM medium. Agr functionality and biofilm formation indicated below the *S. aureus* strain name corresponds to axenic conditions in ASM.
